# A High-Resolution Power Grid Frequency Dataset from Five Solar Sites Across England in April 2023

**DOI:** 10.1038/s41597-026-07651-z

**Published:** 2026-06-23

**Authors:** Maitreyee Dey, Clarke V. Simmons

**Affiliations:** 1 London South Bank University, 103 Borough Road, London, SE1 0AA United Kingdom; 2 Neuville Grid Data, London, SE1 7PB United Kingdom

## Abstract

Frequency is the pivotal indicator of power grid stability as it conveys the instantaneous balance between electrical supply and load demand. Around the world, the power frequency standard is either 50 or 60 Hz and maintaining it within a very narrow range is vital for reliable system operations. This paper presents the Power Grid Frequency Data (PGFD): a high-resolution UK grid frequency dataset collected from five solar sites across England over one month (April 2023). Unlike typical grid frequency data recorded at one-second intervals, this dataset captures frequency dynamics every 10 ms for a 100x improvement in temporal resolution. The dataset contains 1.3 billion data records, and it will help researchers: detect anomalies, transient events, frequency oscillations, assess frequency stability, better understand grid behaviour, and improve frequency control strategies. By making this dataset openly available, this work aims to support the energy transition to a low-carbon emissions future.

## Background & Summary

Power frequency is the pivotal control parameter for alternating current (AC) electrical power grid systems. Commonly referred to as grid frequency, electrical frequency, AC frequency, or line frequency, it is nominally either 50 or 60 Hertz (Hz), depending on geographic region. Maintaining power frequency within a narrow (typically ±1%) tolerance is essential to proper electrical network operation. Frequency stability is a major operational concern for system operators, as frequency deviations and instability can disrupt performance, cause outages, and even damage equipment^1,2^.

Grid frequency is inherently linked to the dynamic balance of electricity supply and load demand. When power generation exceeds demand, the frequency increases; conversely, when demand exceeds generation, frequency drops. Any imbalance will cause a shift in frequency until a new equilibrium is reached.

The time derivative (*df*/*dt*) of frequency provides the Rate-of-Change-of-Frequency (RoCoF), and it conveys how severe the imbalance is. Certain types of electrical protection devices and grid controls are sensitised to RoCoF.

In practice, frequency and RoCoF values are not measured directly but are estimated from sampled voltage waveforms using signal processing techniques such as zero-crossing detection or Fourier-based methods. The accuracy of these estimates is primarily influenced by factors such as sampling rate, noise, and waveform distortion.

In practice, frequency and RoCoF values are not measured directly but are estimated from sampled voltage waveforms using signal processing techniques such as zero-crossing detection or Fourier-based methods. The accuracy of these estimates is primarily influenced by factors such as sampling rate, noise, and waveform distortion. In the United Kingdom (UK), the standard grid frequency is 50 Hz^3^, with an operational range of  ±0.2 Hz and a statutory limit of  ±0.5 Hz above or below. There is also a deadband notch of  ±0.015 Hz around the nominal frequency of 50 Hz within which frequency regulation is not required.

Several methods exist for estimating power system frequency from voltage waveforms^4^. One of the most widely used approaches in practical measurement systems is zero-crossing detection, which is computationally efficient and straightforward to implement^5^. However, zero-crossing methods are sensitive to waveform distortion and noise, which can introduce estimation errors^6^. More advanced techniques, such as Fourier transform-based methods, are computationally more intensive but provide improved accuracy and robustness under non-ideal waveform conditions^7^.

Frequency stability is essential to avoiding operational problems, including brownouts and blackouts. Monitoring and managing frequency is increasingly challenging for grid operators as electrical systems undergo the energy transition to a climate friendly, low-carbon emissions future^8^. Renewable energy generation and load variability, along with network operations and equipment failure, complicate frequency regulation and stability^9^. The increasing integration of inverter-based resources (e.g., wind, solar, EV charging, air-conditioning, and battery storage) reduces system inertia. As a result, maintaining the nominal grid frequency within established limits becomes more challenging

The UK’s new publicly owned National Energy System Operator (NESO) provides frequency data at a temporal resolution of one sample-per-second (SPS) or 1 Hz. Which offers a comparatively coarse view of system stability^10^. Under IEEE performance standard C37.118, Phasor Measurement Units (PMU) can provide finer grained, time-synchronised frequency data.

The Power Grid Frequency Data (PGFD) empirical dataset presented in this paper offers microPMU (microsynchrophasor) derived frequency measurements at 2 samples per AC cycle (i.e. every 10 ms or 100 SPS on a 50 Hz system). Which is 100x higher temporal resolution than NESO’s publicly available data. The time-synchronised data is collected from five solar sites across England. The resulting high-quality dataset contains 8.64 million data records per site per day or a total of 1.3 billion data records across the five sites for the month of April 2023.

Capturing finer-grained, high-precision frequency measurements should enable the detection of subtle network fluctuations and transient events not visible from lower-resolution data. PGFD may help researchers better understand frequency instability and other electrical behaviour. It may also support the development of advanced monitoring, control, and mitigation strategies. By providing high-resolution empirical data, PGFD enables a more granular investigation into the challenges of frequency regulation and contributes to improving grid reliability in an era of low-carbon technology adoption. Supporting fast response frequency support services provided by battery energy storage systems. or investment planning, forensic analysis, and real-time operations.

**Fig. 1:**
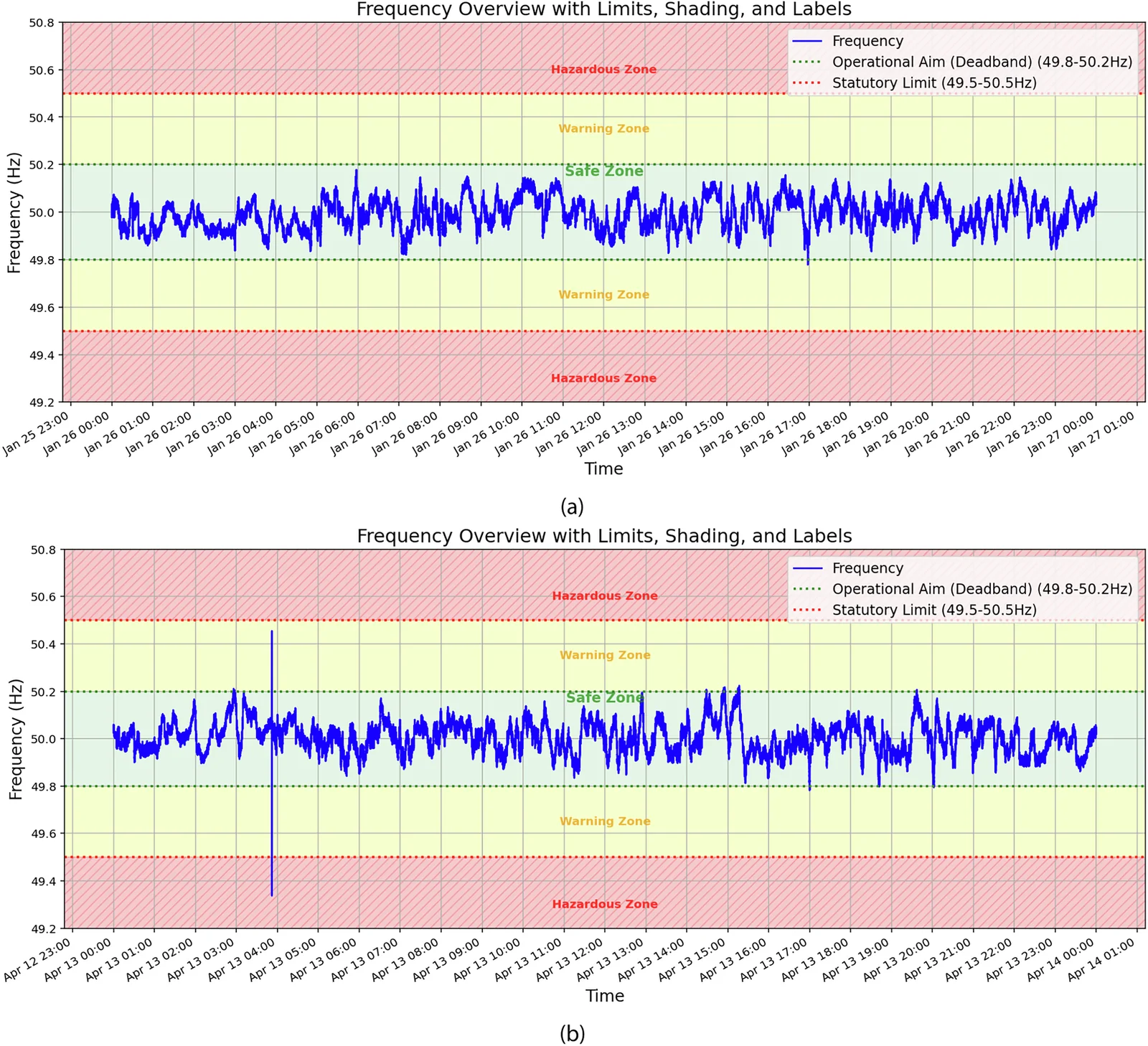
Sample frequency plot: **(a)** without any frequency fluctuations, and **(b)** with frequency fluctuations; from a solar site showing high-resolution daily data from Grid Data Unit (GDU), along with operational aim and statutory limits defined by NESO, the Grid Code and the Electricity Safety, Quality and Continuity Regulations (2002)^32^.

An example of the 10 millisecond-resolution frequency measurements collected over a full day is presented in Fig. 1. The figure illustrates both a representative period of normal frequency behaviour and, on a different day, a clear excursion beyond the statutory operating limits, demonstrating the system’s ability to capture routine dynamics as well as infrequent but operationally significant fluctuations.

With the PGFD dataset, stakeholders can investigate the interplay of grid dynamics, develop anomaly detection methods, and design improved control strategies for a decarbonised grid. By capturing the complexities of real operational environments, this dataset can serves as an open-access resource for advancing power system research in low-inertia, renewable energy-dominated grids^11^.

Previous studies of power system behaviour have often relied on synthetic modelling approaches^12,13^. While such simulations enable controlled experimentation, including the injection of faults and stress-testing scenarios, they may not fully capture the complexity and variability of real-world grid dynamics^14^.

Open-access, high-resolution PMU-derived empirical frequency measurements remain limited^15^. This lack of accessible, high-quality datasets constrains the development and validation of advanced grid analytics and diagnostic tools, particularly given that real-world disturbances are infrequent and often poorly documented^16,17^. In addition, sensor malfunctions or communication failures during critical events can introduce gaps and inconsistencies in recorded measurements.

Several existing initiatives provide access to power grid frequency measurements. GridRadar^18^ provides real-time frequency monitoring services, while the Frequency Monitoring Network (FNET)^19^ enables synchronised frequency measurements for research applications. Public platforms such as power-grid-frequency.org^20^ also offer openly accessible frequency data streams, primarily at second-level resolution. However, these resources typically provide lower temporal resolution or limited spatial coverage compared to the high-resolution, site-distributed dataset presented in this work.

Recent research has turned to data-driven machine learning (ML) and other artificial intelligence (AI) techniques^21^. Deep learning and hybrid approaches have been employed to detect and classify frequency anomalies, with a focus on precise feature extraction and temporal modelling. However, the performance and generalization of AI/ML models depend heavily on the quality, realism, and resolution of the training data^22,23^. The availability of empirical datasets like PGFD should therefore prove helpful to developing data-driven AI/ML toolsets.

April 2023 represents a relevant and representative period for analysing UK grid frequency dynamics. According to the National Energy System Operator (NESO), approximately 46% of Britain’s electricity generation during this period was derived from zero-carbon sources. On April 20th, UK solar generation reached a then record high of 10.971 GW, while total electricity demand for the month was approximately 21 TWh, with peak load occurring on April 5th at around 18:00 h^24^. These conditions reflect a modern, renewable-rich power system, providing an appropriate context for interpreting the high-resolution frequency measurements presented in this dataset.

In summary, PGFD delivers a unique, high-resolution view of UK grid frequency dynamics under modern renewable-dominated operating conditions. By providing access to frequency measurements across five geographically dispersed sites, this dataset offers a valuable resource for understanding grid behaviour, analysing frequency stability, and developing advanced data-driven monitoring and control strategies.

## Methods

Neuville Grid Data has been operating advanced electrical condition monitoring equipment at UK solar farms to improve the facilities’ reliability and performance since late 2019. The equipment is physically attached to the sites’ 11/33 kV distribution grid electrical point of connection. This enables it to simultaneously monitor the solar farm and the wider grid. Thus, providing the basis for collecting PGFD over a wide area.

### Apparatus

Neuville engineers devised a patented apparatus, the Grid Data Unit (GDU)^25^, to collect electrical condition monitoring data. GDUs incorporate a microPMU (also referred to as a *μ*PMU or microsynchrophasor), a power quality monitor (PQM), a Global Positioning System (GPS) receiver for time-synchronisation between sites, and secure bidirectional 3G/4G cellular data telemetry equipment. GDUs are typically installed in a substation. The GDU signal chain starts with analogue voltage and current signals from all three phases. The end result is a comprehensive set of time-synchronised power quality and phasor measurements. Onboard GDU edge-compute capabilities plus reference and buffering data storage support further local analytical processing.

The key GDU component for gathering the PGFD is a PQube3 microPMU from Powerside. The microPMU is compliant with the IEEE C37.118 standard but features much greater measurement precision to meet the needs of distribution network monitoring. With an underlying analogue-to-digital conversion (ADC) at 4 MHz, the microPMU’s digital signal processing (DSP) produces RMS voltage and current phasor values for each phase every half-cycle with a typical Total Vector Error (TVE) of  ±0.01% (which is a 100x improvement on the  ±1.0% TVE of conventional PMUs). On a 3-phase 50/60 Hz installation, the microPMU streams six channels (3 voltage +3 current) at 100/120 data frames per second per channel. These IEEE C37.118 PMU data frames include the GPS time-stamped frequency measurements extracted for the PGFD offering.

### Sites

**Fig. 2:**
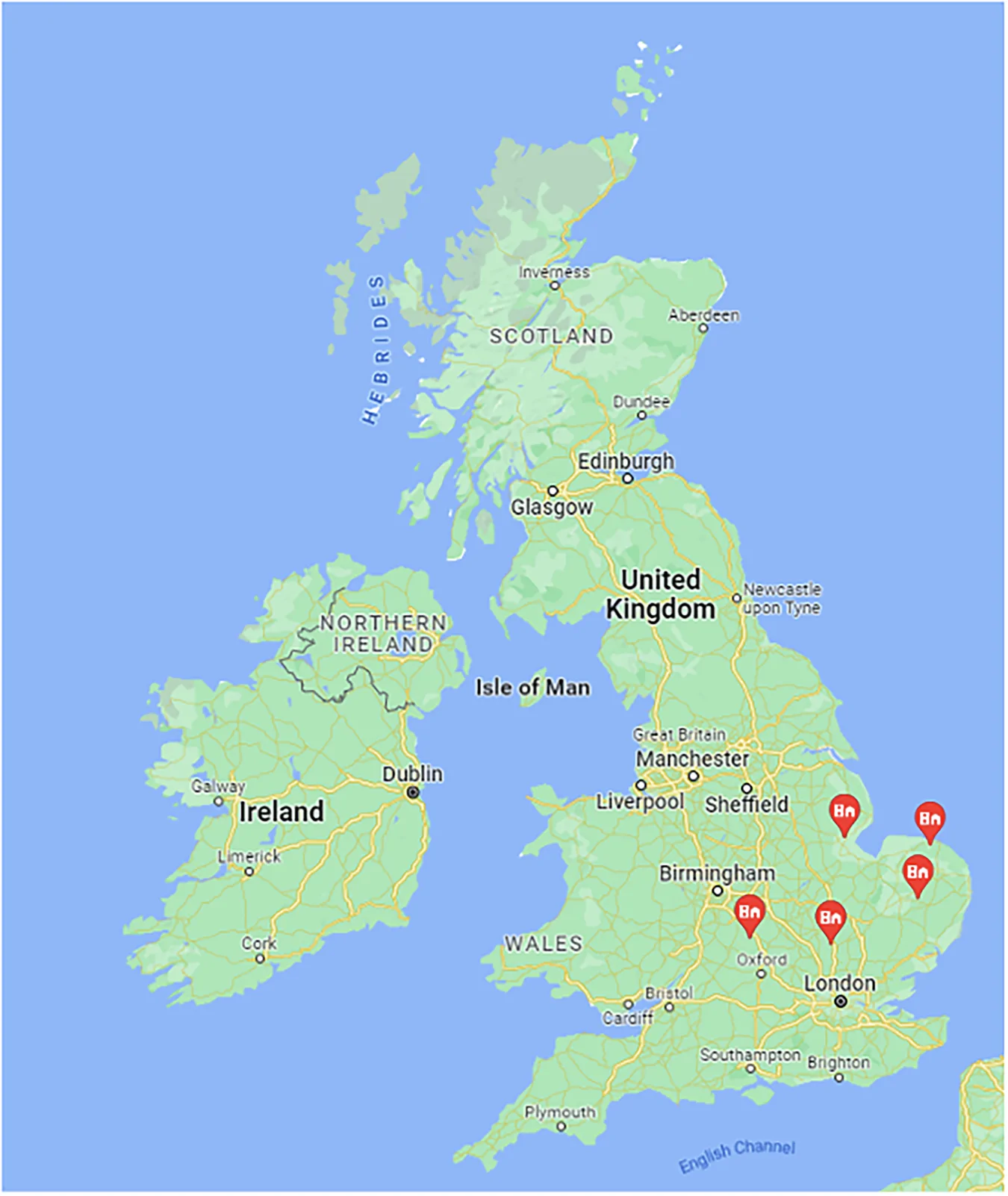
Geographic distribution of solar farm sites across the United Kingdom equipped with GDU devices.

GDUs were installed in five geographically disbursed locations across England – see Fig. 2. These installations enable continuous data acquisition and performance monitoring of the deployed systems. As detailed in Table 1, these five sites are broadly similar, being the same 2014-15 vintage and ranging from 1 to 8 MWp in generation capacity with 11 kV or 33 kV distribution grid connections. Environmental effects across the five sites are also broadly similar, with limited local variation in daily solar irradiance and weather.

**Table 1:** Overview of the solar farm sites, including their locations and operational characteristics.

Site	Name	Location	Site Commissioned	Point of Connection Voltage (kVac)	Inverter Type	Number of Inverters	Generation Capacity (kWdc)
1	Kenninghall	Norfolk	31-Mar-2015	33.0	Central	10	8,000
2	Fen Road	Lincolnshire	28-Jul-2014	11.0	Central	2	1,441
3	Pond Farm	Norfolk	28-Jul-2014	11.0	Central	2	3,605
4	Lodge Farm	Milton Keynes	30-Mar-2014	11.0	String	18	1,010
5	Wroxton	Oxfordshire	31-Mar-2014	11.0	Central	1	1,807

As further detailed in Table 2, these utility-scale (>1 MWp) solar sites are connected to two different local Distribution System Operators (DSO): UK Power Networks (UKPN) and National Grid Electricity Distribution (NGED). NGED was formerly Western Power Distribution (WPD). Table 2 may prove helpful to grid modellers as it provides connective details on how the five sites fit into the wider grid.

**Table 2:** Summary of grid connection parameters for the analysed solar farm sites, including distribution system operator (DSO), export capacity, and associated substation hierarchy.

Site	DSO	Export Capacity (MVA)	Primary Substation	Bulk Supply Point	Grid Supply Point
1	UKPN	12.632	na	Diss Grid 132/33KV	Bramford Grid 132kV
2	NGED	1.263	Sleaford Road Boston 33 11Kv S Stn	Boston 33Kv S Stn	Walpole 132Kv S Stn
3	UKPN	3.158	West Beckham Primary 33kV	Sall Grid 132kV	Norwich Main 132kV
4	NGED	0.900	Wolverton 33 11kV S Stn	Bradwell Abbet 33kV S Stn	East Claydon 132KV S Stn
5	NGED	1.579	Epwell 66 11Kv S Stn	Feckenham 66/11Kv	Feckenham 66 11Kv S Stn

### Data Record

Given its pivotal role in grid balancing and system operation, this paper focuses on offering high-quality frequency data. The dataset comprises microsynchrophasor frequency measurements recorded from five solar sites across England for the month of April 2023^26^. As exampled in Table 3, each PGFD data record consists of a single frequency measurement and its associated timestamp.

**Table 3:** Power Grid Frequency Data (PGFD) time-series database record structure.

Date	Time	Frequency
01/03/2023	06:00:0:000	50.05389
01/03/2023	06:00:0:010	50.05745
01/03/2023	06:00:0:020	50.05549
01/03/2023	06:00:0:030	50.0554
01/03/2023	06:00:0:040	50.05384
01/03/2023	06:00:0:050	50.05265
01/03/2023	06:00:0:060	50.05184
01/03/2023	06:00:0:070	50.0538
01/03/2023	06:00:0:080	50.0547
01/03/2023	06:00:0:090	50.05587

**Fig. 3:**
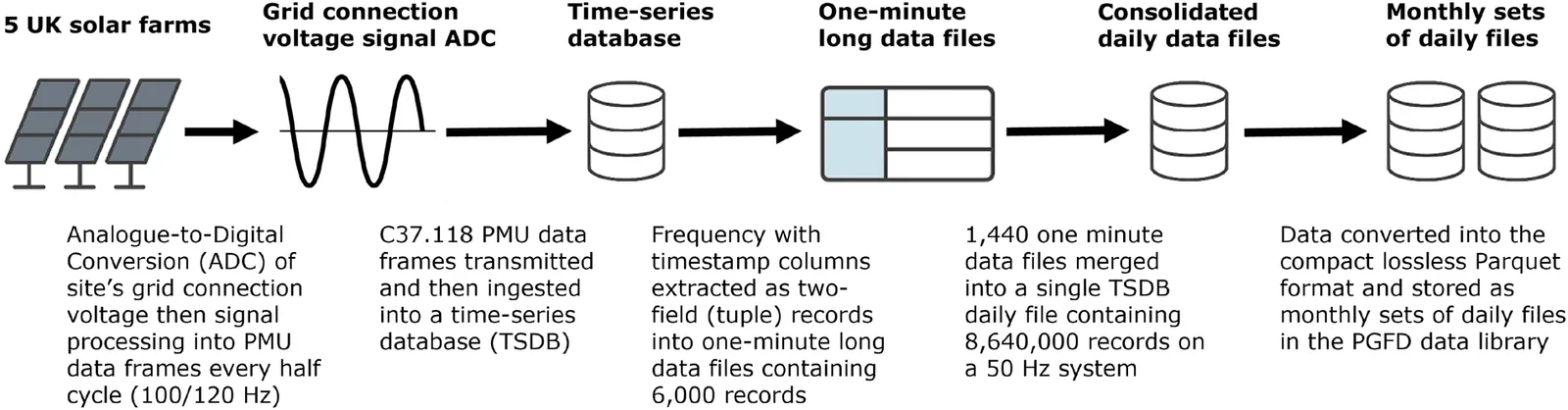
Overview of the data collection and processing workflow.

As shown in Fig. 3 workflow diagram, the frequency data was captured every 10 ms (100/120 Hz) and timestamped accordingly. This comparatively high temporal resolution allows for the detection and tracking of dynamic events. All timestamps are geosynchronised via GPS among sites and recorded in Greenwich Mean Time (GMT).

The two-field (tuple) data records (data-time, frequency) were first collected in a raw binary format with a separate 6,000 row file for each minute of data collection. Then 1,440 one-minute files were combined into a single file per day, containing a total of 8.64 million rows. Each row corresponds to a single frequency measurement captured precisely every 10 milliseconds. From the full set of recorded parameters, only the frequency estimate was extracted and saved into a new, merged PGFD file for further analysis.

Given the large volume of data and its big-data characteristics, the final dataset was stored in the more efficient, lossless compressed Parquet columnar format. Each site’s directory contains 30 daily Parquet files, making it straightforward for researchers to access, manipulate, and analyse the data.

This data processing workflow was applied consistently across all five solar sites and repeated for each day of the target month.

### Ancillary Data

Ancillary NESO system frequency data files are also provided as an adjunct to the PGFD to facilitate comparative research and further cross-validation. They are relatively low-resolution with a one-second sampling rate and provide a single UK system-wide number. Twelve monthly NESO datasets are provided for the entire month of April for the years 2014 through 2025 (https://doi.org/10.6084/m9.figshare.30745268). April 2023 being the direct companion to PGFD. As exampled in Table 4, each NESO frequency record contains three fields: “Date”, “Time”, and “Frequency” for date-time and frequency, respectively. Each of the twelve 72.3 or 87.8 MB files contains 2,592,000 NESO one-second data records per 30-calendar-day month.

**Table 4:** NESO frequency data record structure.

Date	Time	Frequency
01/03/2023	06:00:00	50.052
01/03/2023	06:00:01	50.056
01/03/2023	06:00:02	50.057
01/03/2023	06:00:03	50.057
01/03/2023	06:00:04	50.054
01/03/2023	06:00:05	50.051
01/03/2023	06:00:06	50.048
01/03/2023	06:00:07	50.046
01/03/2023	06:00:08	50.042
01/03/2023	06:00:09	50.037

The full set of NESO frequency data from 2020 onward can be found online at^27^. The NESO archive of pre-2020 frequency data can be found online at^28^.

### Live Streaming Data

Alternately, live UK grid frequency data feeds are publicly available online from NESO and Elexon (Elexon manages Great Britain’s electricity markets). NESO’s website updates its system data every minute with a 4 minute delay – (internal NESO network control systems rely on more granular, near real-time information)^29^. Elexon’s rolling data stream is updated every 2 minutes with a 15 second reporting rate^30^.

## Technical Validation

### Pairwise Correlation

**Fig. 4:**
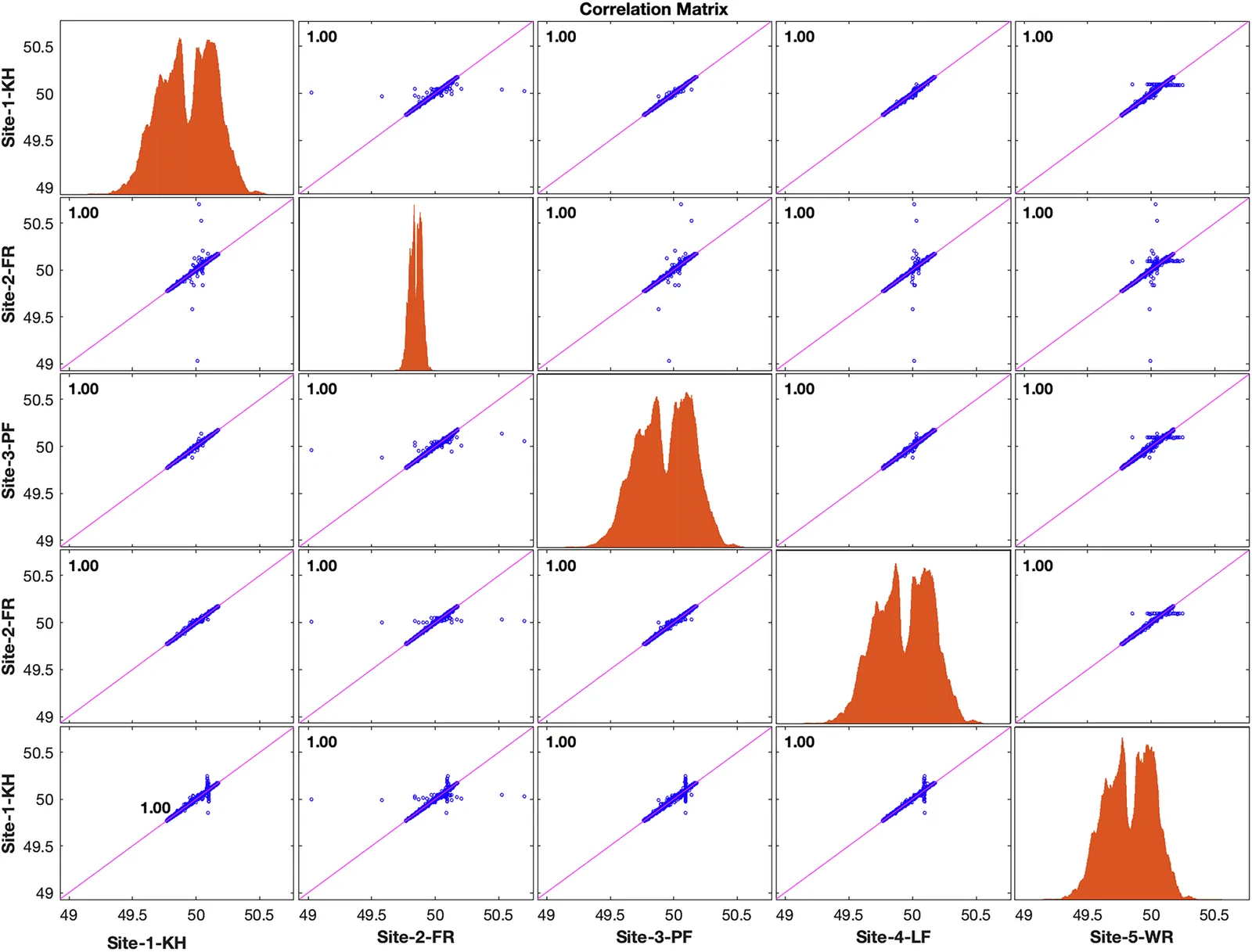
Statistical summary and inter-site correlation analysis of one-day frequency measurements obtained from Grid Data Units (GDUs) across the five solar farm locations.

To cross-validate the raw frequency measurements, Fig. 4 provides a classic “pairwise” correlation matrix (pairplot). It shows a consistent relationships among the five locations: Site 1 (Kenninghall, Norfolk), Site 2 (Fen Road, Lincolnshire), Site 3 (Pond Farm, Norfolk), Site 4 (Lodge Farm, Milton Keynes), and Site 5 (Wroxton, Oxfordshire).

The five sites are plotted in an identical format. On the diagonal are each site’s marginal distribution (orange histogram) with a bimodal spread near 49.7 and 50.3 Hz. Off the diagonal are pairwise scatter plots (blue dots) with the identity line *y* = *x* in magenta. In each panel, the bold number is the Pearson *r*. Every off-diagonal cell reads *r* = 1.00, and the blue scatter clouds collapse tightly onto the *y* = *x* line. The diagonal histograms likewise generally align in shape and location, centring on the same twin peaks near 49.7 and 50.3 Hz.

As expected, these sites move almost in lock-step with regard to their grid connection power frequency. All five exhibit generally the same distribution and inter-site correlation to within machine precision. Thus validating both the measurement accuracy of the GDU and the homogeneity of the UK distribution network around its nominal 50 Hz for the particular day in question. Overall, this demonstrates reproducibility and consistency.

Although the five sites follow an almost identical behavioural pattern, Site 2 (Fen Road) exhibits a noticeably narrower and more tightly centred frequency distribution compared with the other four locations. While the remaining sites show the characteristic bimodal structure around 49.7 Hz and 50.3 Hz, Site 2’s histogram is more compact, with reduced spread and fewer excursions toward the distribution tails. This indicates that, for the particular day analysed, the Fen Road site operated with exceptionally stable frequency measurements, remaining well within the operational target range for a larger proportion of the day than the other sites. This subtle distinction reinforces the sensitivity of the GDU sensors in capturing site-specific behaviour, even when overall inter-site correlations remain effectively perfect.

### Comparison with NESO data

To further validate the consistency of the PGFD dataset, a comparison was conducted with the NESO system frequency measurements for the same sample period. The NESO data provides a lower-resolution (1-second) system-wide frequency reference, which enables external validation of the high-resolution PGFD measurements.

**Fig. 5:**
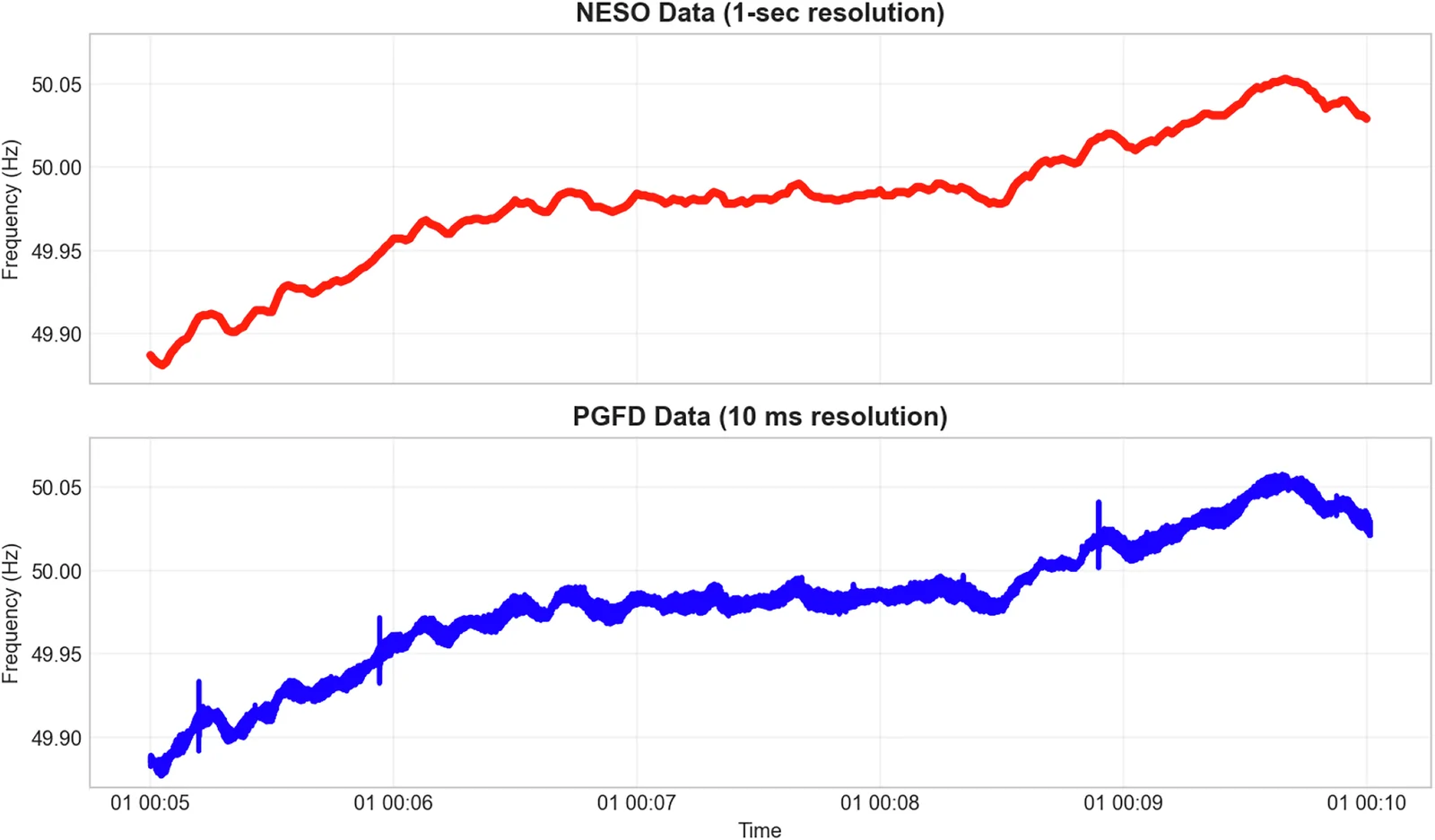
Comparison of grid frequency measurements over the same time window from NESO (top, 1-second resolution) and PGFD (bottom, 10 ms resolution). While both datasets exhibit consistent overall trends, the higher temporal resolution of PGFD reveals fine-scale fluctuations not observable in the NESO data.

Figure 5 presents a representative comparison between the NESO and PGFD datasets over a 5-minute interval in 1^*st*^ April 2023. Both datasets exhibit consistent overall frequency trends and excursions, indicating strong agreement at the system level. However, the higher temporal resolution of PGFD (10 ms) reveals additional fine-scale fluctuations and short-duration variations that are not observable in the NESO data.

For quantitative validation, the PGFD data were downsampled to a 1-second resolution to enable direct alignment with the NESO measurements. The comparison results are summarised in Table 5.

**Table 5:** Quantitative comparison between downsampled PGFD (1-second) and NESO frequency measurements over a representative 5-minute interval.

Metric	Value
Pearson Correlation Coefficient	0.999
RMSE [Hz]	0.0016
MAE [Hz]	0.0013

The results demonstrate excellent agreement between the two datasets, with a near-unity correlation coefficient and very low error values. This confirms that the PGFD dataset accurately captures system-level frequency behaviour, while its higher sampling rate provides additional insight into fine-scale temporal dynamics not present in lower-resolution measurements.

### Comparative Data

The ancillary lower-resolution NESO April 2023 frequency data can be cross compared with Neuville’s higher-resolution PGFD for the same period.

### Weather

Weather plays an important role in power system load demand, renewable energy generation, and network operations. The month of April 2023 was selected as representative for the region. According to Epping Weather News, “April [2023] was an almost exactly average month. There were no extremes of temperature, rain or wind, and the mean values were - well, average”^31^.

## Usage Notes

The PGFD dataset is provided in structured CSV format, with separate files for each measurement site and day. Each record contains timestamped frequency measurements at a resolution of 10 ms, enabling high-resolution time series analysis.

The data can be readily accessed and processed using common programming environments such as Python (e.g., pandas) and MATLAB. Due to the high temporal resolution, users may consider downsampling or windowing techniques for specific analytical tasks.

Potential applications of the dataset include frequency stability assessment, anomaly detection, event classification, and the development of data-driven models for low-inertia power systems. The accompanying ancillary NESO dataset may be used for system-level comparison and validation purposes.

Users should be aware of the high sampling rate and corresponding data volume when performing large-scale analysis.

## Data Availability

The dataset comprises 30 daily frequency data files for five solar sites, including measurements from April 2023. These files are archived in our PFGD database and are accessible at https://doi.org/10.6084/m9.figshare.c.6809259.v1.

## Code availability

A reference Python script for loading, viewing, and inspecting the dataset is available in a publicly accessible GitHub repository. This script was developed and tested using Spyder Version 4. It is a free and open-source Python Integrated Development Environment (IDE). It can be used to locate, load, and visualise the data with minimal setup.

The code is available at: https://github.com/deymaitreyee/parquet-viewer.git. To use it, simply download the dataset, run the script, and follow the prompts to specify the path to the stored files. This approach allows researchers to quickly access and explore the dataset within their preferred Python environment.
